# DNA extraction from hydatid cyst protoscolices: Comparison of five different methods

**DOI:** 10.14202/vetworld.2018.231-234

**Published:** 2018-02-22

**Authors:** Afshin Barazesh, Bahador Sarkari, Sepideh Ebrahimi, Mehdi Hami

**Affiliations:** 1 Student Research Committee, School of Medicine, Shiraz University of Medical Sciences, Shiraz, Iran; 2 The Persian Gulf Tropical Medicine Research Centre, Bushehr University of Medical Sciences, Bushehr, Iran; 3 Basic Sciences in Infectious Diseases Research Center, Shiraz University of Medical Sciences, Shiraz, Iran; 4 Department of Parasitology and Mycology, School of Medicine, Shiraz University of Medical Sciences, Shiraz, Iran; 5 Technical Deputy of East-Azarbaijan Province, Veterinary Directorate, Iran Veterinary Organization, Iran

**Keywords:** DNA extraction, hydatid cyst, protoscolices

## Abstract

**Aim:**

The current study aimed to find out a simple, practical and high throughput DNA isolation method for extraction of DNA from hydatid cyst samples.

**Materials and Methods:**

Cattle and sheep isolate of hydatid cysts were obtained from the slaughterhouse, and hydatid fluid and protoscolices were collected in a sterile condition. Protoscolices were washed, 3 times with phosphate buffered saline, and DNA was extracted by different methods including manual extraction with freeze/thawing and phenol-chloroform, Triton X-100 extraction, and by a commercial kit (YTA, Yekta Tajhiz Azma, Iran) with three different modifications in the kit’s manufacturer instructions. The obtained DNA from the different methods was evaluated by Nanodrop in terms of the yield of DNA and carbohydrates or protein contaminations. To compare the quality of the extracted DNA, two pieces of the mitochondrial genome of *Echinococcus granulosus*, cox1, and nad1, were polymerase chain reaction (PCR)-amplified, using each of the DNA prepared by different methods. Electrophoresis of PCR products was carried out on the agarose gel.

**Results:**

The DNA extracted by manual method, using phenol/chloroform, had the highest yield, yet with the highest level of protein and carbohydrate contamination. The DNA extracted using two-step incubations, initially at 60°C for 2 h and then overnight at 37°C, was the most purified DNA with the lowest rate of contamination.

**Conclusion:**

Findings of the study demonstrated that modification in the currently available commercially DNA extraction kit resulted in the development of a high throughput DNA isolation method. This method can be recommended for the extraction of DNA from hydatid cysts, especially the cattle isolate where the extraction of DNA in these samples are usually problematic.

## Introduction

Cystic echinococcosis (CE) or hydatid cyst is one of the most important zoonotic parasitic diseases. The disease is caused by the infective larval stage of *Echinococcus granulosus* [1]. The adult form of the parasite lives in the intestine of canids as definitive hosts. Humans and herbivore animals, as intermediate hosts, can be infected by ingesting the parasite’s eggs. Hydatid cyst is an important debilitating disease in humans, which often affects vital organs such as liver and lungs [2]. It also makes significant economic losses in livestock [2-4]. Human hydatid cyst has been reported from all countries in the Middle East with a wider endemicity in Iran, Iraq, and Turkey [1,4-7]. About 1% of surgeries conducted at medical centers in Iran are due to hydatid cyst [3].

*E. granulosus* includes a complex of different strains with significant variations in the molecular features as well as their life cycle [2,8-11]. Therefore, determination of the genotypes of the parasite in different CE-endemic areas is of particular importance. On the other hand, to integrate and incorporate information related to morphological taxonomy, molecular genetics, and evolutionary ecology of parasites, the knowledge and a better understanding of biodiversity among these organisms are needed.

Amplification of a specific fragment of DNA by molecular methods is the main step in subsequent genotype analysis, molecular-based diagnosis approaches and taxonomic studies of a given parasite including *E. granulosus* [10,12-16]. The first and fundamental step to achieve these goals and also to identify the dominant genotype of the parasite in a region is the extraction of pure genomic DNA with significant yield. The current study was designed to find out a simple, practical, and high throughput DNA isolation method for extraction of DNA from hydatid cyst samples.

## Materials and Methods

### Ethical approval

The study protocol was approved by Ethical Committee of Shiraz University of Medical Sciences.

### Sample preparation

Lung and liver fresh hydatid cysts of cattle and sheep were obtained from the slaughterhouse and transferred to Parasitology Lab, Faculty of Medicine at Shiraz University of Medical Sciences and fertile cysts were selected. Hydatid fluid and protoscolices were collected in sterile condition into 50 mL Falcon tubes. Protoscolices were washed, 3 times, with phosphate buffered saline (PBS) by centrifugation at 1000 g for 6 min. Then, DNA was extracted from the collected protoscolices by different methods.

### DNA extraction using the commercial kit

Using a commercially available DNA extraction kit (YTA, Yekta Tajhiz Azma, Iran), DNA was extracted by three different ways with modifications to the kit’s manufacturer protocol. About 200 mg of protoscolices sediment was homogenized with 2 mL of PBS, and 100 µL of the suspension was placed in three separate microtubes, and lysis buffer and proteinase K were added to each microtube. The first microtube was incubated initially at 60°C for 2 h and then overnight at 37°C (first method). The second microtube was stored at 60°C just for 2 h (second method), and the third microtube was just incubated overnight at 37°C (third method).

### DNA extraction by manual method using phenol/chloroform and freeze/thawing

Parasite protoscolices were placed in a microtube and 300 µL of lysis buffer was added. The sample was frozen and thawed, 5 times, using liquid nitrogen and water bath. Proteinase K (8 µL) was added to the sample and incubated overnight at 37°C. At the end of incubation, 100 µL of phenol/chloroform was added to the sample and centrifuged for 5 min at 16500 g. The upper clear phase was cautiously transferred into a new microtube and was diluted with the same volume of absolute ethanol. Sodium acetate (1%, 3 Mol) was added to the sample and stored at -20°C, overnight. Then, the sample was centrifuged for 5 min at 16500 g and the supernatant was discarded. The tube was drained upside down to remove the alcohol. Finally, 100 µL of distilled water was added to the tube and vortexed thoroughly.

### DNA extraction using Triton X-100

Parasite protoscolices (100 µL) were mixed with the same volume of Triton X-100 (2%) in a microtube and incubated for 45 min at 70°C. Then, 200 µL of lysis buffer and 20 µL of proteinase K were added to the microtube and incubated for 2 h at 60°C. The rest of the procedure was carried out according to the kit manufacturer’s instructions.

### Determination of the DNA concentration

The yields of extracted DNA as well as the possible contamination of carbohydrates or proteins in all of the DNA samples, extracted by different methods, were evaluated by Nanodrop (Thermo, USA).

### Polymerase chain reaction (PCR) and Gel Electrophoresis

Cox1 and nad1 fragments of parasite mitochondrial genomes were targeted and PCR-amplified using primers JB3 (F): (5’-TTT TTT GGG CAT CCT GAG GTT TAT-3’) and JB4.5 (R): (5’-TAA AGA AAG AAC ATA ATG AAA ATG-3’) for cox1 and JB11 (F) (5’-AGATTCGTAAGGGGCCTAATA-3’) and JB12 (R) (5’-ACCACTAACTAATTCACTTTC-3’) for nad1.

The PCR program for the amplification of both genomic pieces was: 1× (5’ 95°C) + 40× (45” 94°C +35” 51°C + 45” 72°C) + 1× (10’72°C). PCR products were separated on 1.5% agarose gel, using Tris, acetate and EDTA buffer and gel red and the obtained bands were visualized and recorded by a UV detector (Bio-Rad, USA).

## Results

The concentrations of DNA, obtained by different five methods, were measured by Nanodrop and the results are shown in Table-1.

**Table-1:** Comparison of extracted DNA from hydatid cyst by five different methods.

Methods*	DNA concentration (ng/µL)	Protein contamination (absorbance 260/280 nm)	Carbohydrate contamination (absorbance 260/280 nm)
1	24.5	1.8	1.75
2	16	1.6	1
3	10.8	1.7	0.9
4	75.4	1.1	0.5
5	14.5	1.4	0.65

* 1: Samples extracted by the commercial kit in two-step incubation procedure (initially at 60°C for 2 h and then overnight at 37°C); 2: Samples extracted by the commercial kit in one step at 60°C for 2 h; 3: Samples extracted by the commercial kit in one step at 37°C with overnight incubation; 4: Samples extracted manually, using phenol/chloroform and freeze/thawing; 5: Samples extracted by Triton X-100.

**Figure-1:**
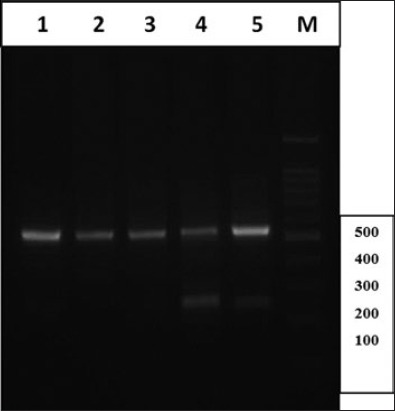
Electrophoresis of polymerase chain reaction products of the nad1 gene of *E. granulosus* using DNA extracted by different methods. Lane 1: Samples extracted by the commercial kit in two-step incubation procedure (initially at 60°C for 2 h and then overnight at 37°C); Lane 2: Samples extracted by the commercial kit in one step at 60°C for 2 h; Lane 3: Samples extracted by the commercial kit in one step at 37°C with overnight incubation; Lane 4: Samples extracted manually, using phenol/chloroform and freeze/thawing; and Lane 5: Samples extracted by Triton X-100; M: Molecular weight marker.

**Figure-2:**
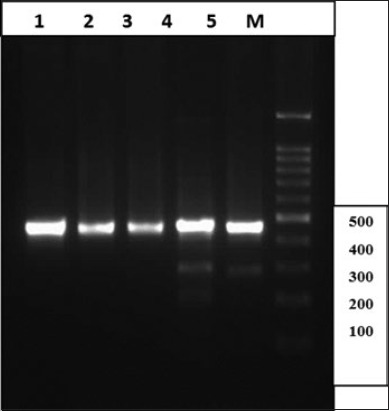
Electrophoresis of polymerase chain reaction products of the cox1 gene of *E. granulosus* using DNA extracted by different methods. Lane 1: Samples extracted by the commercial kit in two-step incubation procedure (initially at 60°C for 2 h and then overnight at 37°C); Lane 2: Samples extracted by the commercial kit in one step at 60°C for 2 h; Lane 3: Samples extracted by the commercial kit in one step at 37°C with overnight incubation; Lane 4: Samples extracted manually, using phenol/chloroform and freeze/thawing; and Lane 5: Samples extracted by Triton X-100; M: Molecular weight marker.

It was found that the DNA extracted by modified commercial kit with two-step incubation procedure (initially at 60°C for 2 h and then overnight at 37°C) had the lowest rate of contamination whereas the extracted DNA by manual method, using phenol/chloroform, had a very high level of contaminations. The extracted DNA was used in a PCR reaction, targeting two mitochondrial genes (cox1 and nad1) of *E. granulosus*. Agarose gel electrophoresis of PCR products revealed the 450 bp fragment of cox1 and a 550 bp fragment of nad1 of *E. granulosus* in all of the samples, extracted by different methods. However, prominent, clean and sharp bands of PCR products were seen in DNA extracted by modification in the commercial kit, using two-step incubations. Figures-1 and 2 show the gel electrophoresis of PCR product of nad1 and cox1 of *E. granulosus*, using DNA extracted by different methods.

## Discussion

One of the common basic methods used in molecular studies for the extraction of DNA is using the phenol/chloroform followed by freeze and thawing. Chloroform and particularly phenol are hazardous chemicals. Phenol is easily absorbed through the skin which can cause severe burns to the skin or may even cause systemic poisoning. Chloroform is highly volatile, and its long-term inhalation exposure may cause central nervous system depression. Moreover, chloroform is a suspected human carcinogen.

During the past decades, different procedures have been developed for the extraction of DNA from biomedical samples. Sharbatkhori *et al*. [17] compared five different methods for DNA extraction from *E. granulosus* protoscolices for PCR-amplification and noted that using liquid nitrogen for freezing and thawing is a simple procedure but is laborious and costly.

Removal of proteins from the extracted DNA by ethanol precipitation is a key step during the phenol/chloroform extraction procedures. In a study by Sung *et al*. [18], to remove the 16s and 23srRNA from the extracted RNA, the researchers have boiled the sample with Triton X-100, and the quality of the purified RNA has been satisfactory. In the present study, to evaluate the effect of Triton X-100 on the quality of the extracted DNA, samples were initially treated with TritonX-100. The results showed that this treatment had no substantial effects on removing the protein and carbohydrates contaminations of the sample.

Findings of the current study demonstrated that utilizing of two incubation conditions (first 2 h at 60°C and then overnight, at 37°C), resulted in the best-extracted DNA in terms of purity and reduction of protein and carbohydrate contaminations. In line with this, the most prominent and sharp band of PCR product on gel electrophoresis was seen in the samples which were extracted by this method.

## Conclusion

In this study, we developed a high throughput DNA isolation method by modification in the incubation time of a commercially available DNA extraction kit. In view of these findings, it can be suggested that this method can be effectively used for the extraction of DNA for hydatid cysts, especially the cattle isolate where the extraction of DNA is usually problematic.

## Authors’ Contributions

BS and AB: Conceived and designed the study, assisted with data analysis and drafted the manuscript; MH: Made contribution to sample collecting and assisted with data analysis; SE and AB: Carried out the experiment and assisted with data analysis. All authors read and approved the final version of the manuscript.
